# Effectiveness of a Digital Awareness App in HIV/AIDS Mitigation Among Transgender Individuals in Rawalpindi District: Protocol for a Quasi-Experimental Study

**DOI:** 10.2196/84610

**Published:** 2026-02-18

**Authors:** Muhammad Mudassar Farooq, Shaheer Ellahi Khan, Ali Ahmed

**Affiliations:** 1 Department of Public Health Health Services Academy Islamabad Pakistan; 2 School of Medicine Division of Infectious Diseases and Global Public Health University of California, San Diego San Diego, CA United States

**Keywords:** HIV/AIDS, digital awareness, mobile app, HIV treatment and prevention, key populations, transgender individuals

## Abstract

**Background:**

HIV/AIDS is a disease associated with stigma and discrimination. This can hinder the adoption of preventive and treatment methods, especially in vulnerable populations, such as the transgender community.

**Objective:**

The primary objectives of this study are to explore awareness barriers related to HIV/AIDS, develop and pilot a mobile-based HIV awareness app, and evaluate its acceptability and usability within the transgender community.

**Methods:**

The research will employ a quasi-experimental design, utilizing a pre- and posttest comparison between an intervention group that will use the mobile app and a comparison group that will not. Phase 1 involves a situational analysis, including key informant interviews, focus group discussions, and a cross-sectional survey. An app will be designed and developed in Phase 2. Phase 3 will comprise a preintervention assessment recruiting 150 transgender people, implementation of the app on the cell phones of 75 transgender people, and a postapp assessment. Statistical techniques will be employed to analyze the captured data and assess the effectiveness of the app.

**Results:**

The recruitment began on August 25, 2025, for the first phase, with the subsequent phases to follow. The data collection and analysis will be completed and finalized by August 31, 2026, following the intervention deployment. No funding was received from any external source for this study.

**Conclusions:**

The results of this study will reveal the effectiveness of a mobile app for the transgender community. These results will determine the continuation and further scale-up of this intervention. The findings will create evidence to inform favorable strategies for vulnerable populations.

**International Registered Report Identifier (IRRID):**

DERR1-10.2196/84610

## Introduction

### Overview

The purpose of this study is to promote HIV/AIDS awareness, especially among key populations such as transgender people, helping not only in this high-risk population but also the public adopt prevention methods and reduce the stigma associated with this disease. An estimated 42.3 million people lived with HIV/AIDS globally in mid-2024, among whom 0.33 million were estimated to live in Pakistan [1]. The National AIDS Control Program (NACP) registered 74,619 people living with HIV at 94 antiretroviral treatment (ART) centers across Pakistan, out of which 51,821 are currently adherent to receive medicine as of December 31, 2024 [2]. The Joint United Nations Programme on HIV/AIDS (UNAIDS) has set ambitious “95-95-95 targets” [1] to reduce the transmission of HIV by the end of 2030. Achieving these targets is challenging with the current strategies, as progress shows [3], particularly due to a lack of proper awareness about the disease and prevention methods—not only in the general population but also in the target populations. The 95-95-95 HIV testing, treatment, and viral suppression targets aim to close gaps in HIV treatment coverage and outcomes [4]. According to these targets, 95% of all people living with HIV globally should have a diagnosis, 95% of people diagnosed should be taking lifesaving ART, and 95% of people on treatment should achieve a suppressed viral load by the end of 2025 [5]. Globally, these percentages were 86%, 77%, and 72% in 2023, respectively. In Pakistan, these statistics are low—by the end of 2022, only 25.8% knew their status, 16.6% were receiving ART, and 6% were virally suppressed [3]. A comprehensive awareness of the causes of HIV may result in reducing new infections in the country.

A rapid rise in HIV prevalence among the transgender community was observed during the latest Integrated Biological and Behavioral Surveillance (IBBS) round (for 2023 and 2024) [6], as compared to the previous survey held in 2016 and 2017 [7]. Recent annual infection trends among key populations show that transgender individuals are more likely to spread infection and are not easily traceable [8]. Viable strategies like condom use and pre-exposure prophylaxis to reduce new infections are necessary [9]. However, awareness and knowledge of the disease are missing components. Even high school students involved in sexual activities without preventive measures put themselves at risk of HIV transmission [10]. No effective solution has been suggested to reduce the risk and adopt strong preventive measures for adolescents. Pakistan continues to experience a rising number of new cases each year from all key and general populations due to a lack of awareness of HIV and a low number of testing services [11]. The government can reduce HIV cases by regulating health care practices, promoting public awareness, supporting counselling, and minimizing transactional sex work [12]. People should be aware of HIV, and access to health facilities should be readily available [13]. However, studies in the country context have not specified how HIV awareness programs should be implemented. Districts including Multan, Chiniot, Sargodha, Rawalpindi, and Dera Ghazi Khan have the highest HIV infection cases [14]. Training health care workers can help reduce stigma and discrimination toward people living with HIV [15]. However, no studies have been found that provide an absolute intervention for reducing the stigma-related factors. Psychological interventions, such as therapeutic alliance and empathy, can improve treatment adherence [16], but techniques for implementing these interventions remain inadequate. Limited household income and a lack of formal education are the main reasons for delayed HIV treatment initiation [17].

Although hospitals provide free testing and treatment for people living with HIV, supported by the Global Fund and the Government of Pakistan [18,19], factors like social stigma, forgetfulness, drug side effects, anxiety, depression, substance abuse, and financial difficulties are the reasons behind poor adherence to ART [20,21]. Only 58% of the registered patients follow up on their treatment in Pakistan, which is a low ratio [22]. Data from the NACP show that 69% of people living with HIV were following their treatment as of September 2024 [23]. Depression is one of the major causes of nonadherence to ART [24,25]. Telehealth improved treatment adherence in people living with HIV, showing a direct relationship between adherence and the quality of their lives [20,26]. Phone call interventions for people living with HIV can help them with treatment adherence, which is a good telehealth technique, but this intervention may not address the stigma around HIV [27]. Patient management, including individual and group counselling, may improve adherence at ART centers [28,29]. Feeling depressed or overwhelmed from daily dosing and a desire to forget about having HIV are the main reported reasons for missing appointments [30]. This study also showed that employed patients are more careful than unemployed patients about their medication. In a review of digital apps for people living with HIV, usability and cost were shown to be hindrances in the African context, despite their effectiveness [31]. Digital interventions for people living with HIV have positively affected adherence to treatment, prevention, and social and behavioral issues [32].

Researchers do not formally publish the studies conducted in the health sector of Pakistan, particularly interventional study designs [33]. Due to a lack of scientific evidence, it is difficult to determine the effectiveness of such interventions. Despite efforts to raise awareness and reduce stigma and discrimination related to HIV/AIDS, barriers to prevention, testing, and treatment—particularly among transgender individuals in Pakistan—are prevalent. Digital interventions can provide a confidential and accessible means to bridge the gaps in preventive measures.

A web-based digital platform, namely Sehat Dost [34], was recently launched but does not specifically address key populations, especially transgender individuals. The proposed awareness mobile app in our study is mobile-based and linked with the web to allow frequent information updates, which will help remove the sociocultural and health care access barriers for key populations at risk of HIV. Audiovisual content in this proposed app will make it easy for people without formal education to understand and gain relevant knowledge and awareness. HIV is a sensitive issue associated with stigma and discrimination, and the existing platforms do not adequately protect user privacy. The proposed app will adopt enhanced data protection protocols. The existing digital information focuses on testing rather than awareness and behavior change, whereas HIV prevention is a main component that needs to be addressed. This study hypothesizes that a mobile-based awareness intervention will enhance knowledge about HIV/AIDS in the transgender community.

### Theoretical Framework

The Health Belief Model, originally developed in the 1950s and updated into the 1980s by Rosenstock [35], is widely used to predict health behaviors. Our digital awareness app will be designed to address this model’s constructs. Informational content in the app may increase perceived susceptibility and severity by providing real-life examples. Practical tips and access to services may reduce perceived barriers and enhance self-efficacy, as presented in Table 1.

**Table 1:** Conceptual table linking the Health Benefit Model constructs to the proposed app’s features and outcome indicators.

Perceived Construct	App Feature	Output
Susceptibility	Risk quiz	Awareness about personal risk
Severity	Testimonials	HIV seriousness understanding
Benefits	Information on prevention and testing	Motivation for testing, prevention, and treatment
Barriers	List of health facilities, FAQs^a^	Reduces fear and misconceptions
Self-efficacy	Quiz, guide	Increases confidence in adapting to prevention
Cues to action	Reminders and alert messages	Participation increase

^a^FAQ: frequently asked question.

Intervention theories of change consider factors that predict behaviors and propose general mechanisms of behavior change [36]. The theory of change will follow a pathway from input to impact trail. App development, content creation, and community engagement are the input variables, whereas digital outreach, awareness messaging, and feedback loops are core activities. The number of users reached, content viewed, and user feedback will be outputs of the intervention. Improved knowledge of the community, increased awareness, and a positive attitude will be considered as short-term outcomes of the mobile-based app, whereas assessing stigma reduction, behavior change, and safer sex practices requires a longer period. Reduction in risky behaviors and new HIV infections presents a long-term impact of this digital intervention. With this pathway in mind, this study focuses on measuring knowledge and awareness as the short-term changes. A 3-to-4-month period of implementation, as proposed in this study, is useful for short-term changes, whereas an extended period is required for bigger behavioral changes. The theory of change will provide a useful lens to interpret the findings. These findings will guide future interventions.

### Rationale

In the current digital era, there is a need to introduce digital strategies for delivering disease-related messages and guiding people, especially high-risk communities, on how to prevent disease. Transgender and other communities, including men who have sex with men (MSM), female sex workers, and people who inject drugs, are among underserved communities that need special attention to improve awareness of HIV disease, as they are at higher risk due to behavioral factors.

### Objectives

This research aims to improve the well-being of transgender individuals through digital intervention in increasing awareness and promoting the adoption of preventive measures against HIV. More specifically, we have the following objectives: (1) to explore existing awareness and barriers regarding HIV/AIDS in the transgender community through situational analysis; (2) to develop and pilot a mobile app for awareness and stigma-related factors associated with HIV among the transgender community in Rawalpindi district; and (3) To evaluate the acceptability and utilization of a digital tool in improving public health awareness.

## Methods

### Overview

**Figure 1:**
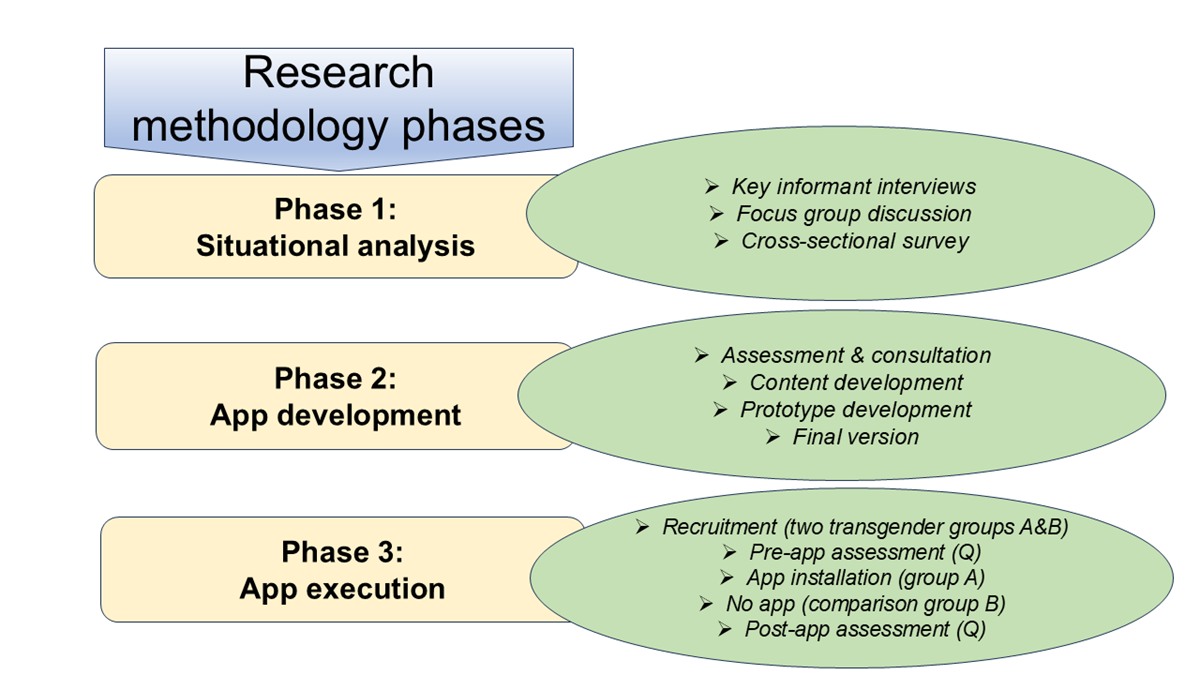
Flow diagram of a quasi-experimental study on HIV mitigation in the transgender community of Rawalpindi, Pakistan, conducted from August 2025 to August 2026.

This research will comprise 3 phases: situational analysis, intervention design, and execution and evaluation. Figure 1 shows an overview of the research design. A transgender leader working closely with the community, a government official from the HIV program, and an IT expert who is an app developer have been consulted and will be on board throughout the study. This board will provide guidance through all study phases.

### Phase 1: Situational Analysis

#### Explanation

The main objective of situational analysis is to explore existing awareness and barriers regarding HIV/AIDS among the transgender community. Identifying the key stakeholders is an important step in situational analysis, wherein concerned national and international organizations like the Association for Social Development, the Association of People Living with HIV, UNAIDS, the World Health Organization (WHO), the Dareecha Male Health Society (DMHS), and the Sathi Foundation will be contacted to explore the ideas and ground realities. Trends and locations/hotspots will be acquired from the NACP and DMHS. Purpose sampling techniques are suitable for key informant interviews and focus group discussions [37,38], whereas snowball sampling for a cross-sectional survey [39] will be adapted in this phase of the study.

#### Key Informant Interviews

In this component, 10 key persons, including transgender community leaders, health care providers, and nongovernmental organization (NGO) representatives, will be engaged to gain an understanding of the challenges and barriers related to HIV/AIDS awareness and health care access. The participants will be categorized into transgender leaders (4 interviews), government officials (2 interviews), United Nations stakeholders, and NGOs (2+2 interviews). A purposive sampling technique will be used to contact the interviewees. Open-ended questions will be asked related to community knowledge, behaviors, HIV services and policies, and adaptation of the digital intervention.

#### Focus Group Discussion

Two groups of transgender individuals will be engaged to explore information related to behaviors and mobile apps. These groups will be selected from key community members in the Rawalpindi district using purposive sampling. The first group will comprise 5 participants aged between 16 and 30 years, and the second group will have a similar number of participants aged 30 years and up. Based on the findings of this focus group discussion, a questionnaire will be developed to administer a cross-sectional survey, which will help us gain a better understanding of the community’s lived experiences.

#### Cross-Sectional Survey

A small sample size cross-sectional survey will be conducted by recruiting 50 transgender individuals as a fixed sample from hotspots in the Rawalpindi district, with data collected through a snowball sampling technique at a single time point. Several cross-sectional studies have been conducted with sample sizes ranging from 30 to 50 as pilot or exploratory works [40,41]. This practice will assess the basic variables, including mobile phone availability and the knowledge, attitudes, and behaviors of the transgender community. The sample size is enough to estimate the preliminary study outcomes. A small sample size is more suitable in studies involving sensitive diseases [42]. The questionnaire will be tested with 10 individuals with similar characteristics to ensure clarity.

#### Thematic Analysis

Thematic analysis will be applied to the qualitative data obtained from the key informant interviews and focus group discussions to extract deep insights and ensure that the digital intervention is designed based on real needs and experiences. Descriptive statistics, including percentages, mean, and median, will be applied to the cross-sectional survey data. Furthermore, chi-square tests and regression analysis will be used to explore associations between knowledge levels and demographic factors. This analysis will establish our baseline understanding for designing an effective digital intervention.

### Phase 2: Intervention Development

#### Explanation

The second phase of research will focus on designing and developing an intervention, a new mobile-based app. The following steps will be taken in the second phase of the study.

#### Assessment and Consultation

Based on Phase 1 and its findings, a mobile app will be developed to provide culturally appropriate, easy-to-understand information on HIV/AIDS prevention, treatment, and testing services. Needs, preferences, messages, and other related material will be consulted with stakeholders. Furthermore, the awareness gap, app language, message format, access to smartphones and the internet, and most importantly, user privacy and confidentiality, will be considered. Health information specialists and IT personnel will also be consulted for designing and developing the app.

#### Content of the App

Content development is the core component of this app, as it can either support awareness or discourage the use of the digital platform on this sensitive issue. A cross-sectional survey from Phase 1 and meetings with key stakeholders will support the development of content for the mobile app. HIV transmission and prevention, ART adherence and treatment literacy, testing and linkage care centers, mental health and stigma support, self-assessment tool, quiz, push notifications, and frequently asked questions (FAQs) are the main modules of this app. Recommendations from key stakeholders and transgender individuals will be valuable for incorporation into the app. The audiovisual content will be designed to be accessible to all users, including those with and without formal education, and will be available in both English and Urdu.

#### Prototype Development

A minimum viable product of the digital intervention will be designed by the IT personnel hired for this purpose and implemented for a small sample size of 10 transgender individuals. Basic content sections, including awareness messages, chatbot, offline access, and adherence support, will be included. This pilot will give feedback about the security, compatibility, and user-friendliness of the app.

#### Final Version

At this stage, feedback from a small-scale test will be integrated to develop the final version of the mobile app. The app developer will fix bugs, optimize app speed, and ensure compatibility with Android devices. A manual for this app will also be developed along with back-end data protocols.

### Phase 3: Intervention and Evaluation

#### Explanation

The third phase of research will comprise the intervention execution. The following methods will be adopted.

#### Study Design

A quasi-experimental study will be adopted to evaluate the effectiveness of the digital intervention on HIV awareness among key populations, particularly the transgender community. Propensity score matching (PSM) will be applied to create statistically similar baseline groups for the intervention and nonintervention categories. This technique will be used for group allocations to reduce bias, not as a sampling method. The difference-in-differences technique will be used to evaluate the effectiveness of the digital HIV awareness app. The app will not be installed in the cell phones of the comparison group during the observation period, although other routine services will be provided accordingly. Both groups will be monitored during the intervention period to ensure data authenticity.

#### Area and Duration of the Study

Two groups of transgender individuals from Rawalpindi district will be enrolled with their consent; participants will have some awareness of HIV and engage in behaviors that place them at risk of contracting HIV. They will be interviewed, and data will be collected using a structured questionnaire. Then, the digital app will be installed on the cell phones of the intervention group. The facilitator will provide 1 hour of hands-on training to the intervention group, scheduled in convenient sessions, regarding the use of the app. After 3 months, the same questions will be asked, and any changes in their behavior will be observed across both the intervention and comparison groups. During the first month of this phase, a baseline survey will be conducted to assess existing awareness levels in both the intervention group and the comparison group. The intervention phase will last for approximately 3 months, during which participants in the intervention group will actively engage with the app's educational content. A follow-up survey will be conducted with the same participants within 1 month. This will measure changes in awareness and compare the results between the 2 groups. The final month will be devoted to data analysis and presentation of findings for review.

#### Data Sources

Data from the national program will be obtained to review current estimates and the registration status of people living with HIV. The hotspots will be identified based on patient burden in the area. Primary data will be collected from the transgender community of Rawalpindi for quantitative analysis.

#### Study Population

Preliminary results from IBBS (round 2023-2024) showed a 2.8% increase in transgender individuals compared to the 2016-2017 round, along with increases of 1.5% in MSM and 1.6% in female sex workers and a 10.6% decrease in people who inject drugs [6,7]. This indicates that the transgender community is less likely to adopt preventive measures compared to other key populations. Rawalpindi, Kasur, Sheikhupura, Sialkot, and Hyderabad are cities with a high number of new cases among transgender individuals in 2024 [2]. The registered cases at the DMHS are residents of Rawalpindi, while community-based organizations (CBOs) in other high-burden districts also report transfer-in cases from various cities. Therefore, transgender individuals from high-risk areas of Rawalpindi will be selected based on predefined criteria. Transgender individuals from Rawalpindi will be recruited from different hotspots, CBOs, and outreach services. A purposive sampling technique applying the PSM method will be used.

#### Technique and Size Calculation

A purposive sampling technique will be used, and PSM will be applied to balance the 2 groups for a more valid comparison. A standard power of 80% and a 95% confidence level will be used to minimize errors, and the expected effective size will be set at 0.5. High statistical power will be used, which means an 80% chance of detecting the impact of awareness that is high. A moderate effect size (*d*=0.5) means that the expected impact of the digital app on HIV/AIDS awareness is noticeable and meaningful but not extreme. This level suggests that, on average, the intervention group will score 0.5 standard deviations higher than the comparison group. It is assumed that σ=1 for the sample size calculation, using it as an assumption, not as a measured value. The exact standard deviation will be found after collecting baseline data.

The sample size formula resulted in 63 individuals in each group for the study. Considering the dropout scenarios, 75 participants will be recruited.

The sample size calculation: where



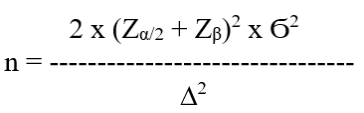



Z_α/2_ = *z* score for the chosen confidence level (eg, 1.96 for 95% confidence)

Z_β_ = *z* score for power (eg, 0.84 for 80% power)

Ϭ = estimated standard deviation of the outcome variable, assumed 1

∆ = expected difference between group means (effect size × standard deviation)



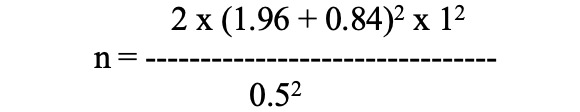





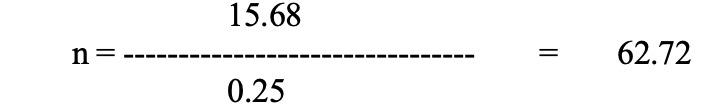



Sample size = 63

#### Sample Recruitment

This study will recruit the participants into 2 groups based on certain inclusion and exclusion criteria, particularly applicable to the intervention group. The eligibility criteria are as follows: (1) transgender individuals aged 18 years and above; (2) residents of Rawalpindi; (3) own a smartphone; and (4) are willing to install the mobile app. The participants must have sufficient digital literacy to use the app and provide informed consent. Individuals who have prior training in HIV/AIDS awareness programs, those unwilling to provide consent, and those lacking basic operational literacy for smartphone apps will be excluded.

#### Data Collection Tool

A structured questionnaire will be developed and adapted based on coordination, meetings, and formal interviews of the key stakeholders working in the HIV community. WHO assessment tools will also assist in preparing the questionnaire. This primary data collection tool will include basic demographics, multiple-choice questions, and Likert scale items to evaluate the participants’ baseline and postintervention knowledge, attitudes, and perceptions about HIV/AIDS. Consent forms for each participant will include thumbprints and signatures.

#### Data Collection Techniques

A structured questionnaire will be developed and administered to participants to gather data for this study. This questionnaire will be given to both intervention and comparison groups to assess baseline knowledge of HIV/AIDS before app installation and to measure changes in awareness after the intervention. The survey will be conducted using well-designed forms to ensure efficient data collection. Community members will be recruited through outreach, and the 2 groups will be evaluated for effectiveness in a quasi-experimental design [43]. Participants will be recruited through a community-based outreach program and collaboration with key stakeholders involved in the HIV and health sectors. A pretest of the questionnaire will be conducted with a subset of the population to ensure its reliability and clarity. Any ambiguities or difficulties in the questionnaire will be addressed before it is fully deployed. One month will be allocated to gather baseline information and conduct meetings before installing the app. Participants will be recruited from community-based organizations, homes, gatherings of key populations, and some designated health centers. Measures will be taken to ensure consistency and reduce bias throughout the process.

#### Pretest or Pilot Study

The structured questionnaire will be administered to a subgroup of 10 people who meet the same inclusion and exclusion criteria to assess clarity, relevance, and ease of understanding. The results of this pilot study will help to refine the study design, ensuring that questions are unambiguous, the intervention is effective, and the data collection process runs smoothly. Necessary modifications will be made before launching the full study.

#### Reliability and Validity

Both the questionnaire and digital intervention will be pretested in a pilot study with 20 participants of similar characteristics to ensure consistent understanding. A test-retest method will check consistent responses from the subset of participants at different time intervals. The scores from both times will be compared on the Pearson correlation coefficient (*r*), with values greater than 0.6 considered acceptable for reliability. The Cronbach alpha will be used to measure the internal consistency of the questionnaire. This technique is commonly used to assess internal consistency, and expert reviews will ensure content validity [44]. Cronbach alpha is considered more reliable than other methods for measuring internal consistency [45]. If a reliability test yields an α ≥ 0.7 based on full baseline data, this will indicate that our tool is suitable. The WHO HIV knowledge assessment tool will also be considered when preparing the questionnaire. Pre- and posttest scores will be analyzed to validate whether the digital intervention effectively improves awareness.

#### Data Analysis Plan

Descriptive and inferential statistical methods will be applied to the collected data to assess the effectiveness of the digital intervention in improving HIV/AIDS awareness. Descriptive statistics, including mean, standard deviation, frequency, and percentage, will be used to summarize the participants’ basic demographic characteristics. Pre- and posttest scores within the intervention group will be compared using paired *t* tests. Independent *t* tests will be used to compare outcomes between the study groups. PSM will be applied to control for baseline differences between the 2 groups. Cronbach alpha will be calculated to measure internal consistency and evaluate the reliability of the questionnaire. A test-retest method will be applied, and the Pearson correlation coefficient will assess the stability of the questionnaire over time. Regression analysis will be carried out to identify the factors influencing HIV/AIDS awareness improvement. SPSS and Microsoft Excel (IBM Corp) software will be used for data analysis. The results will be presented in tables, graphs, and charts for interpretation. *P*<.05 will be considered statistically significant.

#### Sustainability

The proposed mobile-based app will be linked to a website to enable content updates. Login details and usage information stored on the website will be used to measure app performance and success. A feedback survey on the app content will help guide improvements. This app will be monitored on various indicators, including number of app downloads, user registration or logins, session frequency and duration, content completion rates, clickstream data, activity participation, push notification response rates, feedback submitted, and repeat usage or return rate.

### Expected Outcomes

The targeted community is expected to gain an improved understanding of HIV and its transmission. The participants will become more aware of prevention methods, treatment access, care, and support services. Relevant, accessible, and inclusive content will enhance community engagement in digital public health initiatives. It is anticipated that knowledge and behavior-based indicators will be improved. Positive feedback from stakeholders will indicate readiness to scale up this intervention.

### Ethical Considerations

This research was approved by the Ethical Review Board and Graduate Research Management Council of Health Services Academy (00022/HSA/PhD-2022) on July 22, 2025. The consent form, available in both English and Urdu, will cover 2 sections, including information and consent. Participant confidentiality will be strictly upheld, and their data will be safeguarded. A clear message within the app stating, “your data is encrypted,” will help to build trust and encourage engagement.

## Results

Ethical Review Board approval was obtained on July 22, 2025. Recruitment of the 10 key informant interviews occurred between August 25 and 30, 2025, followed by the focus group discussion on September 8, 2025. Recruitment of the 50 transgender people for the cross-sectional survey took place from October 1 to 20, 2025. The app development process occurred between December 1, 2025, and January 5, 2026. The recruitment of 150 participants and the app installation process for 75 participants will be completed by February 28, 2026, followed by an assessment period until June 14, 2026. A follow-up survey will be conducted from June 15 to 30, 2026, and the report will be completed on August 31, 2026. Pre- and postintervention surveys will be compared using the appropriate statistical methods. This analysis will determine the intervention's effectiveness. The study results will provide a foundation for policymakers to serve at-risk and underserved communities. Given the sensitivity of stigma-related disease, understanding the community context is important for carrying out any intervention. Descriptive statistics will guide us about the demographics of the participants, and inferential statistics will give us key information about awareness levels and behaviors. The results will be displayed in both tables and graphs to provide a clear understanding of awareness levels and changes over time in both the intervention and control groups.

## Discussion

### Expected Findings

It is anticipated that a mobile-based app for the transgender community in Rawalpindi will increase community awareness and guide them to access prevention and treatment services. The culturally sensitive content of this app will enhance the participation and acceptability level among transgender individuals on digital platforms. The intervention group is expected to become more aware of the disease and its prevention and treatment methods as compared to the control group. App users are also expected to share HIV-related knowledge with other community members and guide them to adopt preventive measures by avoiding risky behaviors.

Studies on digital platforms for marginalized communities show some improvement in their desired objectives. The availability of studies for transgender individuals in the country context is limited. Therefore, specific evidence is not available in this context about the success of the interventions. However, findings from available studies helped build the concept of our study. The results of this study will generate evidence on the use of mobile apps within the transgender community. Insights and cultural aspects of the transgender community will support the development of policies for their well-being.

Limited HIV awareness among the key population, particularly transgender individuals in Pakistan, highlights the need for a digital app considering stigma sensitivity. HIV is an urgent and sensitive public health issue that needs to be addressed by introducing new but authentic interventions. This is the first community-based and locally contextual mobile-based app that will improve awareness, encourage HIV testing, and reduce misconceptions about HIV. While a mobile-based app for transgender individuals is a new idea for this community, the rigorous methodology applied in this study will support science-based decision-making.

A mixed methods approach is the main strength of this study, as the situational analysis provides the baseline for the quasi-experimental study of a hard-to-reach community. This research also emphasizes ethical considerations aligned with community norms, culture, and sensitivities, strengthening the expected outcomes. Technological literacy issues may hinder the process, but training participants and audiovisual aids will mitigate such issues. The study findings have the potential to support scale-up of the intervention to other key populations, including MSM and female sex workers.

The results of this study will be disseminated in the first week of September 2026 in a seminar to be held at the Health Services Academy, to which focal people from the NACP, UNAIDS, WHO, CBOs, and other key stakeholders will be invited. Further, the results will be submitted to a peer-reviewed journal for publication.

### Limitations and Mitigation

Recruiting transgender individuals using the probability sampling method is impractical due to hidden hotspots, stigma, discrimination, and cultural aspects. Therefore, this study adopted snowball sampling for the cross-sectional survey. A comprehensive baseline data set on relevant covariates, along with cofounders, will be collected by ensuring sufficient overlap and appropriate matching methods. If PSM does not achieve an adequate balance between the intervention and control groups, the study will proceed with stratified analysis to reduce bias. In this approach, the sample will be divided into homogeneous subgroups called strata based on age group, education, or baseline knowledge. Multivariable regression analysis may also be used to identify any imbalance in covariates, which will isolate the effect of the app from other factors.

### Conclusion

This study is expected to provide insights into improving HIV/AIDS awareness among transgender individuals in Rawalpindi through a mobile app–based intervention. This study also has the potential to support scale-up of the intervention in subsequent phases to achieve improved health outcomes. The findings are expected to guide future planning in the public health sector related to HIV/AIDS, particularly for vulnerable populations. It will also help reduce stigma and discrimination by strengthening community engagement.

## Abbreviations

ART: antiretroviral treatment
CBO: community-based organization
DMHS: Dareecha Male Health Society
IBBS: Integrated Biological and Behavioral Surveillance
MSM: men who have sex with men
NACP: National AIDS Control Program
NGO: nongovernmental organization
PSM: propensity score matching
UNAIDS: Joint United Nations Programme on HIV/AIDS
WHO: World Health Organization

## Data Availability

Data confidentiality and availability will be addressed in the informed consent form. The data will be available upon request after the personal information has been anonymized.
